# Detection and replication of QTL underlying resistance to gastrointestinal nematodes in adult sheep using the ovine 50K SNP array

**DOI:** 10.1186/s12711-016-0182-4

**Published:** 2016-01-20

**Authors:** Marina Atlija, Juan-Jose Arranz, María Martinez-Valladares, Beatriz Gutiérrez-Gil

**Affiliations:** Departamento de Producción Animal, Facultad de Veterinaria, Universidad de León, Campus de Vegazana s/n, León, 24071 Spain; Instituto de Ganadería de Montaña, CSIC-ULE, Grulleros, León, 24346 Spain; Departamento de Sanidad Animal, Universidad de León, León, 24071 Spain

## Abstract

**Background:**

Persistence of gastrointestinal nematode (GIN) infection and the related control methods have major impacts on the sheep industry worldwide. Based on the information generated with the Illumina OvineSNP50 BeadChip (50 K chip), this study aims at confirming quantitative trait loci (QTL) that were previously identified by microsatellite-based genome scans and identifying new QTL and allelic variants that are associated with indicator traits of parasite resistance in adult sheep. We used a commercial half-sib population of 518 Spanish Churra ewes with available data for fecal egg counts (FEC) and serum levels of immunoglobulin A (IgA) to perform different genome scan QTL mapping analyses based on classical linkage analysis (LA), a combined linkage disequilibrium and linkage analysis (LDLA) and a genome-wide association study (GWAS).

**Results:**

For the FEC and IgA traits, we detected a total of three 5 % chromosome-wise significant QTL by LA and 63 significant regions by LDLA, of which 13 reached the 5 % genome-wise significance level. The GWAS also revealed 10 significant SNPs associated with IgA_t_, although no significant associations were found for LFEC. Some of the significant QTL for LFEC that were detected by LA and LDLA on OAR6 overlapped with a highly significant QTL that was previously detected in a different half-sib population of Churra sheep. In addition, several new QTL and SNP associations were identified, some of which show correspondence with effects that were reported for different populations of young sheep. Other significant associations that did not coincide with previously reported associations could be related to the specific immune response of adult animals.

**Discussion:**

Our results replicate a FEC-related QTL located on OAR6 that was previously reported in Churra sheep and provide support for future research on the identification of the allelic variant that underlies this QTL. The small proportion of genetic variance explained by the detected QTL and the large number of functional candidate genes identified here are consistent with the hypothesis that GIN resistance/susceptibility is a complex trait that is not determined by individual genes acting alone but rather by complex multi-gene interactions. Future studies that combine genomic variation analysis and functional genomic information may help elucidate the biology of GIN disease resistance in sheep.

**Electronic supplementary material:**

The online version of this article (doi:10.1186/s12711-016-0182-4) contains supplementary material, which is available to authorized users.

## Background

Persistence of gastrointestinal nematode (GIN) infection and the related control methods have major impacts on the sheep industry worldwide [1]. The extensive use of anthelmintics has negative consequences, such as the costs of treatments, the emergence of anthelmintic-resistant strains of parasites, and the presence of drug residues in animal products. Among different alternatives to chemical control, the selection of genetically-resistant animals has been suggested to reduce dependence on the use of anthelmintics [2, 3]. Selective breeding for resistance to GIN using fecal egg count (FEC) as an indicator trait has been undertaken for certain sheep breeds [4–6]. However, classical selection for this complex phenotype is hindered by the time-consuming and costly process of recording information for indicator phenotypes (which may also include serum levels of e.g., immunoglobulin A (IgA), IgE and pepsinogen) and by the requirement for animals to be infected by GIN at sampling. These difficulties suggest that selecting animals resistant to GIN infection would be more efficient if it was based on indirect estimates, such as those generated from molecular marker information. In the last few decades, considerable effort has been made to understand the relationship between host and parasite and the mechanisms that underlie host resistance [7]. Moreover, the recent availability of the Illumina OvineSNP50 BeadChip (Illumina Inc., San Diego, CA) (referred to here as the “50 K chip”) and a high-quality reference genome assembly [8] may allow for a deeper understanding of the genetic architecture of complex traits in sheep. Effective exploitation of this molecular information will increase our chances of developing protocols that will enable efficient selection of animals with increased resistance to GIN infections.

Because GIN are particularly pathogenic to young naïve animals such as growing lambs, gastrointestinal infections constitute a major cost to the sheep meat industry [9]. Accordingly, most of the quantitative trait locus (QTL) studies on GIN resistance traits [10], including those based on microsatellite markers as well as more recent analyses that exploit the ovine 50 K chip, have been conducted primarily on young animals [11–26]. Conversely, for the Mediterranean dairy sheep industry, a production system that is based on adult ewes and the sale of suckling lambs fed exclusively on maternal milk, replacement ewes and adult sheep are the only animals subjected to the direct effects of helminth infections [27]. In these animals, the breakdown of the acquired immunity to infection that occurs around the time of parturition [28] and the necessity of anthelmintic treatment determine how severe the economic losses will be [29].

Previously, we performed a genome scan using microsatellite markers to identify QTL that influence indicator traits of parasite resistance in adult Churra dairy sheep, an autochthonous dairy breed of the northwest region of Castilla y León in Spain [20]. The lack of strong coincidence between the QTL that we had identified and those previously detected by using lamb data suggested that aside from differences in host-parasite combinations, these QTL could be related to different mechanisms that underlie resistance between adult sheep and lambs.

Within this context, we undertook a new QTL mapping study based on the use of the ovine 50 K chip to genotype a commercial population of Spanish Churra dairy sheep. To follow on the initial linkage analysis-based genome scan reported by Gutiérrez-Gil et al. [20], our study was designed to replicate some of the QTL that were detected by the microsatellite-based scan and to identify new QTL and allelic variants associated with two previously analyzed indicator traits of parasite resistance: FEC and serum levels of IgA. For this purpose, we performed the new analyses using a different set of half-sib families from the same commercial population of Spanish Churra sheep. Taking advantage of the increased marker density offered by the 50 K chip, in addition to classical linkage analysis (LA), we also implemented combined linkage disequilibrium and linkage analysis (LDLA) and genome-wide association study (GWAS) approaches to provide a more complete picture of the QTL that segregate in this ovine population.

## Methods

### Resource population and sampling

Phenotypic and genotypic information for 518 Churra ewes from the Selection Nucleus of the National Association of Churra Breeders (ANCHE) was analyzed. The animals belonged to 14 half-sib families and were produced by artificial insemination, with an average family size of 37 daughters per sire (ranging from 12 to 89). A single collection of fecal and blood samples was performed for each of the 17 flocks in the Castilla y León region where the animals were raised. The samples were later processed to measure two indicator traits of parasite resistance, FEC and serum IgA levels. The ages of the sheep included in this study ranged from 4 to 11 years. At the time of sampling, all the sheep were undergoing milking and were at least in their third lactation.

### Phenotypic records

FEC measurements were determined by floating the feces samples in zinc sulfate (d = 1.33) solution on a McMaster slide and counting the eggs [30]. The detection limit for this technique was 15 eggs per gram (epg). The samples showed a low level of FEC, which was related to the exceptionally small amount of rainfall before and during the sampling period. For each flock, pooled feces were cultured to recover and identify third-stage larvae (L3) using standard parasitological techniques [30]. One hundred L3 were identified per flock to estimate the percentage of each helminth species.

IgA activity in serum was tested against a somatic antigen from the fourth-stage larvae (L4) of *Teladorsagia circumcincta* by indirect ELISA according to a modified protocol that was previously described by Martinez-Valladares et al. [31]. Briefly, ELISA plates (Sigma) were coated overnight with 100 µL of phosphate buffered saline (PBS) solution containing 2.5 µg/mL of *T. circumcincta* L4 somatic antigen. On the following day, the ELISA test was performed in four steps. After each step, the content of the plate was removed, the plate was washed, and each well was filled with a specific reagent; the plates were then incubated for 30 min. The following reagents were used for each step: (1) PT-Milk (4 g powdered milk + 100 mL PBS-Tween 20; PBS-Tween 20: 1 L PBS (pH 7.4) + 1 mL Tween 20 (Sigma)); (2) a sheep serum; (3) a rabbit anti-sheep IgA antibody and (4) a peroxidase substrate and tetramethylbenzidine solution to produce a color reaction that was stopped after 30 min by the addition of 50 μL of 2 M H_2_SO_4_. The results were measured as optical density (OD) values. Positive and negative controls were included in all the plates; positive controls were obtained from a pool of sera from sheep that were experimentally infected with *T. circumcincta* and negative controls were obtained from non-infected sheep that were maintained indoors. The results are expressed as optical density ratios (ODR) according to the following formula:

$$ODR = \frac{{\left( {sampleOD - negativeOD} \right)}}{{\left( {positiveOD - negativeOD} \right)}}$$

### Statistical analyses

Prior to further analyses, FEC measurements were log-transformed (LFEC) to reduce over-dispersion, since no transformation yielding a normalized FEC dataset was available. However, Box-Cox power transformation was used for the IgA phenotype to obtain a normal distribution of values (IgA_t_). We used the R *‘car’* library to estimate the power parameter λ and carry out the transformation [32]; the log-transformation was also calculated through a command line in R [33].

To assess the variables that influence the two parasite resistance-related traits under study, an analysis of variance (ANOVA) was performed for LFEC and IgA_t_ using a general linear model (GLM) through the R command line [33], which included the three following fixed effects: flock, age and time point relative to parturition. The ‘flock’ effect was classified into 17 groups. For the ‘age’ effect, two groups were considered i.e. ewes four to six years old and ewes seven or more years old. For the ‘time point relative to parturition’ effect, two categories were also considered i.e. one that included ewes that had a low immune response possibly because they were in the last stage of pregnancy or beginning lactation (animals sampled 2 weeks before giving birth or 30 days after birth) and one that included ewes that were outside that specific period.

### Genotypes and physical map

We analyzed the genotypes that were obtained with the 50 K chip for a population of 1696 Churra ewes [34], which included animals with available phenotypic measurements for parasite resistance traits. First, SNP order and genome positions were updated according to the latest available version of the ovine Genome Assembly, Oar_v3.1 [35] by considering a 1 cM–1 Mb conversion rate. Then, quality control (QC) of the genotypes was performed for the entire genotyped population according to the protocol described in [34]. Briefly, QC was performed in seven steps that were applied to raw genotypes using the following criteria: (1) a GenCall score for raw genotypes greater than 0.15; (2) known location of the SNPs on the ovine autosomes; (3) a call rate per individual greater than 0.9; (4) a call rate per SNP greater or equal to 0.95; (5) minor allele frequency (MAF) higher than 0.05; (6) a *p* value for Hardy–Weinberg equilibrium (HWE) greater than 0.00001; and (7) analysis of the filtered genotypes using the VerifTyp software to check for Mendelian inconsistencies between parents and offspring (Boichard D and Druet T, personal communication). A total of 43,613 SNPs located on the 26 ovine autosomes passed the QC for the population of 1696 Churra ewes. For these 43,613 SNPs, available genotypes for 518 animals with parasite resistance phenotypes were subjected to different QTL mapping analyses.

### QTL mapping analyses

Yield deviations (YD) of transformed data were used as dependent variables for statistical analyses to identify genomic regions that influence resistance to GIN infection. For the two traits under study, YD estimates were calculated following a multivariate animal model using the R command line and the *‘lsmeans’* library [36]. LFEC and IgA_t_ were corrected for the fixed effect of ‘flock’, which according to the previously described ANOVA analysis, was the only factor that significantly influenced the studied traits. Then, the following statistical procedures were used for QTL mapping:

(1) Genome scans based on a classical LA and a combined LDLA procedure were performed at 0.1 cM step intervals using the corresponding analysis options (*calcul* = 4 and *calcul* = 28) of the QTLMap software [37]. Using this software, we also calculated the significance thresholds at the chromosome-wise significance level through a total of 1000 permutations (at 0.1 cM steps) for LA and 1000 simulations (at 5 cM steps) for LDLA. Genome-wise significance thresholds were based on the chromosome-wise significance threshold by correcting for the total number of chromosomes under analysis. A by-default haplotype size of four SNPs was used for LDLA.

For each QTL identified by the across-family LA scan, linkage-based within-family analyses were performed to identify the corresponding segregating families. For the significant QTL that were detected by LA, likelihood ratio test (LRT) values were converted to logarithm odds ratio (LOD) values [15], and confidence intervals (CI) for the QTL locations were estimated by the widely used 1-LOD drop-off method [38]. The proportion of phenotypic variance that was explained by the QTL detected by LA was calculated based on the corresponding LOD values using the formula $\sigma_{p} = 1 - 10^{{\frac{ - 2}{n}LOD}}$ [39]. In the LDLA, chromosomal regions that involved consecutive significant haplotype associations within a chromosome (allowing gaps no greater than 5 cM) were grouped as a significant LDLA interval and the remaining ones were considered as isolated significant haplotypes.

For chromosomes with significant effects that were identified by both LA and LDLA genome scans, a linkage disequilibrium analysis (LDA) based on the LDA decay approach of Legarra and Fernando [40] was implemented using the QTLMap software (*calcul* = 26). The aim of this analysis was to determine whether the significant associations identified by LDLA were exclusively due to linkage pedigree-related information or whether an association with the trait could also be identified at the population level. Similar to the previously described LDLA, LDA was performed at 0.1 cM step intervals using a by-default 4-SNP haplotype size and 1000 (at 5 cM steps) simulations for the chromosome-wise threshold calculation. Significant LDA intervals were defined in the same way as for LDLA.

(2) A GWAS was performed by implementing the following linear mixed model (LMM), which includes the polygenic effect as a random effect and genotypes at single SNPs as fixed effects: (${\mathbf{y}} = {\rm {\mathbf {Zu}}} + {\rm {\mathbf{X}}}b + e$) where ${\mathbf{y}}$ is defined as the vector of phenotypes (YD) of the ewes; ${\mathbf{Z}}$ is a matrix associating random additive polygenic effects to individuals; **u** is a vector containing random polygenic effects; **X** is a vector with a genotypic indicator (−1, 0, or 1) that associates records to the marker effect; *b* is the allele substitution effect for the analyzed SNP; and *e* is the random residual. This association analysis was implemented by the restricted maximum likelihood (REML) method using the DMU package [41], and the SNP effect was tested using a Wald test against a null hypothesis of *b* = 0.

Bonferroni corrections for multiple-testing were used to estimate the genome-wise and chromosome-wise significant thresholds for the GWAS-based analyses. To account for the existence of linkage disequilibrium (LD) between the analyzed SNPs, rather than performing a conservative Bonferroni correction based on the total number of SNPs analyzed, we implemented the method proposed by Gao et al. [42] to calculate the number of independently analyzed SNPs for each chromosome and for the entire sheep genome. To this end, we used the simpleM test [43], which estimates the actual number of effective tests (Meff) in genome-wide association studies through a principal component analysis (PCA) approach. Using a PCA-cutoff of 0.975, the total number of independently analyzed SNPs across the entire genome was equal to 25,881.

### Comparison with previously reported QTL and identification of functional candidate genes

We performed a systematic search for previously reported QTL and associations related to parasite resistance traits in sheep for which a good correspondence was observed with the significant associations that we identified in our study; in addition, we performed a search for positional candidate genes in relation to our results. However, prior to these searches, for each significant QTL and significant SNP association identified, we determined a “target genomic interval” (TGI), which was defined as the genomic region based on the sheep reference genome assembly Oar_v3.1 that corresponded to: (1) the CI that was estimated for the significant QTL detected by LA and for the defined significant LDLA intervals; and (2) a 250 kb-long interval centered on each of the significant isolated haplotypes detected by LDLA and the significant SNPs identified by GWAS.

Once the TGI were defined, they were compared with the Oar_v3.1 intervals that are annotated in the SheepQTL database (SheepQTLdb) [10] for previously reported QTL and that are mainly derived from microsatellite-based genome scans. We also compared these TGI with more recent data from studies based on the 50 K chip that are not included in this database [21–26]. For some of these recent data based on the sheep genome assembly Oar_v2.0, when available, the corresponding Oar_v3.1 position of the target marker/interval was considered for comparison. Only regions that mapped within 1 Mb from the defined TGI were considered to coincide with our results. For the QTL that covered a very long region, the position of the QTL peak was prioritized to determine a possible correspondence.

The extraction of positional candidate genes included in the TGI according to the sheep genome assembly (Oar_v3.1) was performed using the BioMart web-based tool [44] based on the Ensembl release 81. Functional candidate genes related to the QTL identified in this study were identified by comparing the complete list of positional candidate genes extracted with BioMart with a database of 5029 immune-related genes. This database was based on the IRIS (1535 genes [45]) and ImmPort (4815 genes) gene lists, both of which are available at [46].

## Results

### Phenotypes

The presence of nematodes was confirmed in all the studied flocks with *Trichostrongylus spp.* and *Teladorsagia spp.* being the most prevalent species (49.3 and 48.6 %, respectively) that were identified among the total number of third-stage larvae obtained for the studied population. The prevalence of GIN infection by FEC per flock was 88.2 % (mean = 42.8 epg) and per individual was 45.4 % (mean = 39.4 epg). Faecal egg counts of GIN ranged from 0 to 1290 epg. For individual animals, the mean ODR of the IgA activity was 4.1 and ranged from 0.09 to 32.9.

### QTL regions

The LA genome scan identified three 5 % chromosome-wise significant QTL (Table 1); in contrast, the LDLA genome scan identified 63 significant regions at the 5 % chromosome-wise level (Table 2). The LDA, which was performed for the three chromosomes that showed coincident results between the LA and LDLA scans, supported some of the significant signals that were identified previously (See Additional file 1: Table S1, Additional file 2: Figure S1). Although ten significant SNPs associated with IgA_t_ (Table 3) were identified in the GWAS, no significant associations were detected for LFEC. The significant results are described below and those identified by more than one analysis are highlighted. For ease of comparison, Table 4 provides a summarized representation of the results of the three analyses performed across the entire genome (LA, LDLA and GWAS).

**Table 1:** Significant chromosome-wise QTL detected by linkage analysis (LA)

Trait^a^	OAR^b^	Across-family analysis				Within-family analysis		
		Pos of max LRT (cM)^c^	P_c_-value^d^	CI (cM)^e^ TGI (Mb)^f^	Positional candidate genes involved in immune response^g^	Segregating family identifier (Pos of max LRT)^h^	CI (cM)^i^	Size effect trait units (SD units)^j^
LFEC	6	88.1	<0.05	80.8–91.4	*AFP, ALB, AMBN, AMTN, AREG, BTC, CXCL1, CXCL10, CXCL11, CXCL9, EREG, GC, IGJ, IL8, MUC7, PF4, PPBP, RASSF6, SCARB2, TMPRSS11D*	Fam. 1 (94.9)	70.0–96.8	0.468 ± 0.015 (0.30)
						Fam. 7 (90.6)	80.4–94.8	−0.499 ± 0.012 (0.32)
						Fam. 11 (86.7)	76.4–96.4	−0.777 ± 0.029 (0.50)
	8	2	<0.05	1–3.4	*CD109, COL12A1, MYO6*	Fam. 4 (31.2)	25.4–35.8	1.218 ± 0.024 (0.78)
						Fam. 11 (1.8)	0–2.9	0.738 ± 0.024 (0.47)
IgA_t_	22	3.4	<0.05	0.3–5.8	*PCDH15*	Fam. 8 (6.4)	0.3–9.9	0.527 ± 0.046 (0.68)

^a^Analyzed traits: *LFEC* log-transformed faecal egg count, *IgA*_*t*_ Box-Cox-transformed optical density ratio (ODR) values of immunoglobulin A activity

^b^ *OAR* ovine chromosome

^c, h^Position of the chromosome (in centiMorgans) at which the maximum likelihood ratio test of the LA is reached in the analysis involving the 14 half-sib families included in this work (across-family analysis) or the individual analysis of the segregating families (those showing a P_c_-value <0.05 in the within-family analysis), respectively

^d^ *P*_*c*_ *-value* Chromosome-wise significance P-value established through 1000 permutation analysis

^e,i^ *CI* Confidence interval (in cM) estimated from the position of the max LRT for the across-family analysis and the within-family analyses, respectively, following the 1-LOD-drop-off method [38]

^f^ *TGI* Target genomic interval (Mb) defined as the corresponding genomic region, according to the sheep reference genome assembly Oar_v3.1, to the CI estimated for the LA significant QTL

^g^Positional candidate genes included in the CI of the corresponding QTL that were highlighted by the immune response candidate gene survey performed in the present work as potential functional candidates

^j^Estimated size effect of the QTL identified in the within-family analysis expressed in trait units (Yield Deviations of IgA_t_) and in phenotypic SD of the trait (in brackets)

**Table 2:** Chromosome-wise significant results (P_c_-value <0.01) from the combined linkage disequilibrium and linkage analysis (LDLA)

OAR^a^	Trait^b^	Pos of max LRT^c^ (cM)	Significant LDLA interval (cM)^d^	P_c_-value (P_g_-value)^e^	TGI (Mb)^f^	Positional candidate genes involved in immune response^g^
1	LFEC	136.9	136.9–143	<0.05	136.9–143	*CXADR, NRIP1*
	IgA_t_	242.4	–	<0.05	242.1–242.5	–
2	LFEC	78.3	–	<0.05	78.17–78.36	–
	IgA_t_	188.3	188.01–188.44	<0.05	188.01–188.44	–
3	IgA_t_	159.8	–	<0.05	159.67–160.06	–
		177.7	–	<0.05	177.52–177.89	–
4	LFEC	57.9	54–58	<0.05	54–58	*DOCK4, IFRD1, LRRN3*
	IgA_t_	8.9	–	<0.0019 (<0.05)	8.66–9.49	–
5	LFEC	5.2	–	<0.0019 (<0.05)	5.02–5.43	*FCHO1, JAK3, MAP1S, UNC13A*
		89.9	–	<0.0019 (<0.05)	89.68–90.14	–
6	LFEC	36	–	<0.05	35.84–36.28	–
		72.5	72.3–77.2	<0.0019 (<0.05)	72.3–77.2	–
		89.9	85–90.2	<0.05	85–90.2	*ALB, AMBN, AMTN, ANKRD17, AREG, BTC, EREG, IGJ, IL8, PF4, PPBP, RASSF6*
7	LFEC	22.8	12.65–25.5	<0.0019 (<0.05)	12.65–25.5	*ACIN1, AJUBA, BBS4, CCNB1IP1, CD276, CDH24, CEBPE, CHD8, CIDEB, CMTM5, DAD1, EFS, EMC4, FEM1B, IL25, IRF9, ITGA11, LRP10, LTB4R, MAP2K1, NEO1, NFATC4, NOX5, NPTN, PIAS1, PSMB5, PSME1, PSME2, RIPK3, RNASE2, RNF31, SMAD3, SMAD6, TRAV16, TRAV21, TRAV24, TRAV27, TRAV36DV7, TRAV39, TRAV4, TRAV41, TRAV5, TRDC, TRDV2, TRDV3, UACA, ZNF219, ZWILCH*
		36.8	36.8–37.3	<0.05	36.8–37.3	–
		53.3	–	<0.05	53.08–53.46	*UNC13C*
8	LFEC	2.3	0.3–12.8	<0.05	0.3–12.8	*CD109, COL12A1, IBTK, IRAK1BP1, MYO6, PHIP, SNAP91, TPBG*
		38.3	37.7–39.2	<0.05	37.7–39.2	–
		49.8	49.59–50.04	<0.05	49.59–50.04	–
		64.1	61.1–64.1	<0.05	61.1–64.1	*BCLAF1, CITED2, IFNGR1, IL20RA, IL22RA2, MAP3K5, PERP, TNFAIP3*
		71.4	71.2–73.8	<0.0019 (<0.05)	71.2–73.8	*PPIL4, STXBP5*
9	LFEC	5.8	–	<0.05	5.64–6.03	*PRKAR1A*
		16.9	–	<0.05	16.75–17.16	–
		24.5	–	<0.05	24.34–24.78	–
		41.7	–	<0.05	41.56–41.96	–
	IgA_t_	56.6	55.9–56.6	<0.05	55.9–56.6	*TPD52*
		67.8	63.4–67.8	<0.05	63.4–67.8	*EBAG9*
10	LFEC	71.6	–	<0.05	70.01–71.55	–
	IgA_t_	27.2	21.5–27.2	<0.05	21.5–27.2	*CKAP2, FOXO1, FREM2, POSTN, SMAD9*
		52.9	–	<0.05	52.68–53.06	–
		78.6	–	<0.05	78.39–78.8	*SLC10A2*
11	LFEC	4.2	4.1–4.27	<0.05	4.1–4.27	–
	IgA_t_	51.1	45.4–51.1	<0.05	45.4–51.1	*ACE, ARHGDIA, B3GNTL1, CD7, CD79B, DDX42, ERN1, FSCN2, GCGR, ICAM2, ITGB3, MAP3K3, MRC2, MYADML2, PSMC5, PSMD12, SMARCD2, SMURF2*
12	LFEC	3.6	–	<0.05	3.34–3.84	*IKBKE, IL10, MAPKAPK2*
12	IgA_t_	1.7	–	<0.05	1.52–1.98	*LRRN2, MDM4, NFASC*
		17.7	–	<0.05	17.56–17.96	–
		72.3	69.5–75.4	<0.05	69.5–75.4	*CAMK1G, CD34, CD46, CFHR5, IRF6, LAMB3, TRAF5*
13	IgA_t_	3.7	3.7–6.3	<0.05	3.7–6.3	–
15	IgA_t_	33.6	33.56–33.93	<0.0019 (<0.05)	33.56–33.93	–
		47	47–53.2	<0.05	47–53.2	*ARHGEF17, ARRB1, DNAJB13, FCHSD2, FOLR1, IL18BP, INPPL1, PAAF1, PGAP2, RELT, RPS3, STIM1*
		70.2	70.06–70.47	<0.0019 (<0.05)	70.06–70.47	–
16	IgA_t_	10.5	–	<0.05	10.29–10.74	–
		64.8	63.8–64.8	<0.0019 (<0.05)	63.8–64.8	*SEMA5A*
17	IgA_t_	18.4	14.6–30.1	<0.0019 (<0.05)	14.6–30.1	*ELMOD2, IL15, PCDH10, PCDH18, PLK4, UCP1*
		36	–	<0.05	35.8–36.22	–
		46	–	<0.05	45.85–46.27	*STX2*
		62.3	62–66.8	<0.0019 (<0.05)	62–66.8	*CMKLR1, CORO1C, HPS4, PIWIL3, PLA2G1B, PXN, RAB35, SART3, SPPL3, TRIAP1, UNG, WSCD2*
20	LFEC	4.8	–	<0.05	4.58–5.04	*BMP5*
21	LFEC	8.1	8.07–8.35	<0.05	8.07–8.35	–
		31.8	31.7–32.24	<0.05	31.7–32.24	–
		43.9	43.7–44.03	<0.05	43.7–44.03	*ACTN3, CTSF, SPTBN2*
21	IgA_t_	17.5	16.5–17.5	<0.0019 (<0.05)	16.5–17.5	*GAB2*
		46	45.97–46.25	<0.05	45.97–46.25	*FGF19*
22	IgA_t_	6.7	5.3–7.3	<0.05	5.3–7.3	*MBL2, PCDH15*
		19.5	–	<0.05	19.26–19.85	*NKX2*-*3*
23	IgA_t_	8.3	–	<0.05	8.15–8.47	–
		23.3	23.3–28.5	<0.05	23.3–28.5	*DSC1, DSC2, DSC3, DSG1, DSG2, DSG3, DSG4,*
		33.9	32.8–38	<0.05	32.8–38	*ADCYAP1, COLEC12, EMILIN2, GATA6, LAMA3, MIB1, NPC1, ROCK1, THOC1, USP14*
		45.8	41.7–48.5	<0.05	41.7–48.5	*ATP5A1, CIDEA, PIAS2, PSMG2, RALBP1,SIGLEC15, SKOR2, SLC14A1, SMAD2*
		54.9	54.56–55.06	<0.05	54.56–55.06	*TCF4*
24	LFEC	2.2	1.91–2.65	<0.05	1.91–2.65	*CLDN6, CLDN9, HCFC1R1, TNFRSF12A*
		17.9	–	<0.05	17.68–18.12	*UMOD*
25	LFEC	37	36.89–37.21	<0.0019 (<0.05)	36.89–37.21	–

^a^ *OAR* ovine chromosome

^b^Analyzed traits: *LFEC* log-transformed faecal egg count, *IgA*_*t*_ Box-Cox-transformed optical density ratio (ODR) values of immunoglobulin A activity

^c^Position of the chromosome (in centiMorgans) at which the maximum likelihood ratio test (LRT) is reached in the LDLA

^d^A significant LDLA interval (in centiMorgans) was defined by clustering consecutive significant 5 % chromosome-wise LDLA associations on a chromosome (allowing gaps no greater than 5 Mb)

^e^P_c_-value: chromosome-wise P-value established through 1000 simulations. P_g_-value: genome-wise P-value obtained from the P_c_-values corrected for the total number of chromosomes analyzed

^f^ *TGI* (Mb) Target genomic interval. For each significant LDLA association, target genomic intervals were defined as the genomic region based on the sheep reference genome assembly Oar_v3.1 that corresponded to the defined significant LDLA intervals (for those regions with consecutive significant positions) and a 250-kb long interval centered on each of the significant isolated haplotypes detected by LDLA

^g^Positional candidate genes extracted from the LDLA significant associations (within the significant LDLA interval if identified, or within a ±125 kb interval from the position of maximum LRT-value for the significant QTL based on isolated significant haplotypes) that were identified as potential functional candidate genes in the search for immune-related genes

**Table 3:** Chromosome-wise SNPs significantly associated with the IgA_t_ trait as identified by the GWAS

OAR^a^	SNP name	SNP position (Mb)^b^	Allele substitution effect trait units (SD units)^c, d^	Nominal P-value	Corrected P_c_-value (P_g_-value)^e^	TGI (Mb)^f^
8	OAR8_53084022.1	49,525,147	0.325 ± 0.075 (0.417)	2.04E−05	0.02	49.40–49.65
8	s42819.1	72,402,305	0.190 ± 0.045 (0.243)	3.77E−05	0.037	72.27–72.52
10	s56461.1	17,012,728	0.221 ± 0.050 (0.283)	1.51E−05	0.013	16.88–17.13
10	OAR10_23921485.1	24,187,107	0.203 ± 0.048 (0.260)	2.63E−05	0.022	24.06–24.31
10	s61799.1	30,924,195	0.210 ± 0.051 (0.269)	5.41E−05	0.045	30.79–31.04
11	DU232778_232.1	32,492,623	0.203 ± 0.048 (0.26)	3.74E−05	0.036	32.36–32.61
12	s68938.1	61,866,746	0.233 ± 0.047 (0.299)	1.28E−06	0.001 (0.033)	61.74–61.99
14	OAR14_21336208.1	20,773,096	0.284 ± 0.070 (0.364)	6.75E−05	0.041	20.64–20.89
15	s75729.1	24,870,677	0.266 ± 0.059 (0.341)	8.33E−06	0.007	24.74–24.99
25	s21640.1	13,152,201	0.224 ± 0.056 (0.287)	9.09E−05	0.048	13.02–13.27

^a^ *OAR* ovine chromosome

^b^Position of the significant SNP identified by the GWAS analysis based on the Oar_v3.1 version of the Ovine Genome Assembly (http://www.ensembl.org/Ovis_aries/Info/Index)

^c, d^Magnitude of the allele substitution effect, and standard error, in trait units (Yield Deviations of IgA_t_) and in phenotypic standard deviations (SD) units (in brackets)

^e^Corrected P-values at the 5 % chromosome-wise level (and 5 % genome-wise level) obtained after applying a Bonferroni correction considering the number of independent markers analyzed for each chromosome and for the whole genome, respectively

^f^ *TGI* Target genomic interval defined for the GWAS significant associations as 250 Kb long intervals centered on the significant SNP. The genes within that interval were extracted as positional candidate genes. In this case, none of these genes was identified as functional candidate by the candidate gene survey performed

**Table 4:** Summary of the QTL detected by the three analyses performed in this study

OAR^1^	LA^2^	LDLA^3^	GWAS^4^
1		LFEC_(a)_; IgA_t(b)_	
2		LFEC_(a)_; IgA_t(b)_	
3		IgA_t(a)_; IgA_t(b)_	
4		*IgA*_*t(a)*_; LFEC_(b)_	
5		*LFEC*_*(a)*_ *; LFEC*_*(b)*_	
6	LFEC_(b)_	*LFEC*_*(a)*_; LFEC_(b)_	
7		*LFEC*_*(a)*_; LFEC_(b)_; LFEC_(c)_	
8	LFEC_(a)_	LFEC_(a)_; LFEC_(b)_; LFEC_(c)_; LFEC_(d)_; *LFEC*_*(e)*_	IgA_t(c)_; IgA_t(e)_
9		LFEC_(a)_; IgA_t(a)_; IgA_t(b)_; LFEC_(b)_; LFEC_(c)_; LFEC_(d)_	
10		IgA_t(b)_; IgA_t(c)_; IgA_t(e)_; LFEC_(f)_	IgA_t(a)_; IgA_t(b)_; IgA_t(d)_
11		LFEC_(a)_; IgA_t(c)_	IgA_t(b)_
12		LFEC_(a)_; IgA_t(a)_; IgA_t (b)_; IgA_t(d)_	*IgA*_*t(c)*_
13		IgA_t_	
14			IgA_t_
15		*IgA*_*t(b)*_; IgA_t(c)_; *IgA*_*t (d)*_	IgA_t(a)_
16		IgA_t(a)_; *IgA*_*t(b)*_	
17		*IgA*_*t(a)*_; IgA_t(b);_IgA_t(c)_; *IgA*_*t(d)*_	
18			
19			
20		LFEC	
21		LFEC_(a)_; *IgA*_*t(b)*_; LFEC_(c);_ LFEC_(d)_; IgA_t(d)_;	
22	IgA_t(a)_	IgA_t(a)_; IgA_t(b)_	
23		IgA_t(a)_; IgA_t(b)_; IgA_t(c)_; IgA_t(d)_; IgA_t(e)_	
24		LFEC_(a)_; LFEC_(b)_	
25		*LFEC*_*(b)*_	IgA_t(a)_
26			

^1^ *OAR* ovine chromosome

^2, 3, 4^Significant QTL for the two analyzed traits (*LFEC* log-transformed faecal egg count, *IgA*_*t*_ Box-Cox-transformed optical density ratio (ODR) values of immunoglobulin A activity) identified by the three genome scan performed in the present study, using linkage analysis (LA), combined linkage disequilibrium and linkage analysis (LDLA) and genome-wide association study (GWAS)

^a, b, c, d, e, f^Different subscripts letters indicate that the QTL in the same chromosome are located at more than 5 cM/Mb of distance

QTL in normal characters detected at the 5 % chromosome-wise level

QTL in italic characters detected at the 5 % genome-wise level

#### LA results

**Fig. 1:**
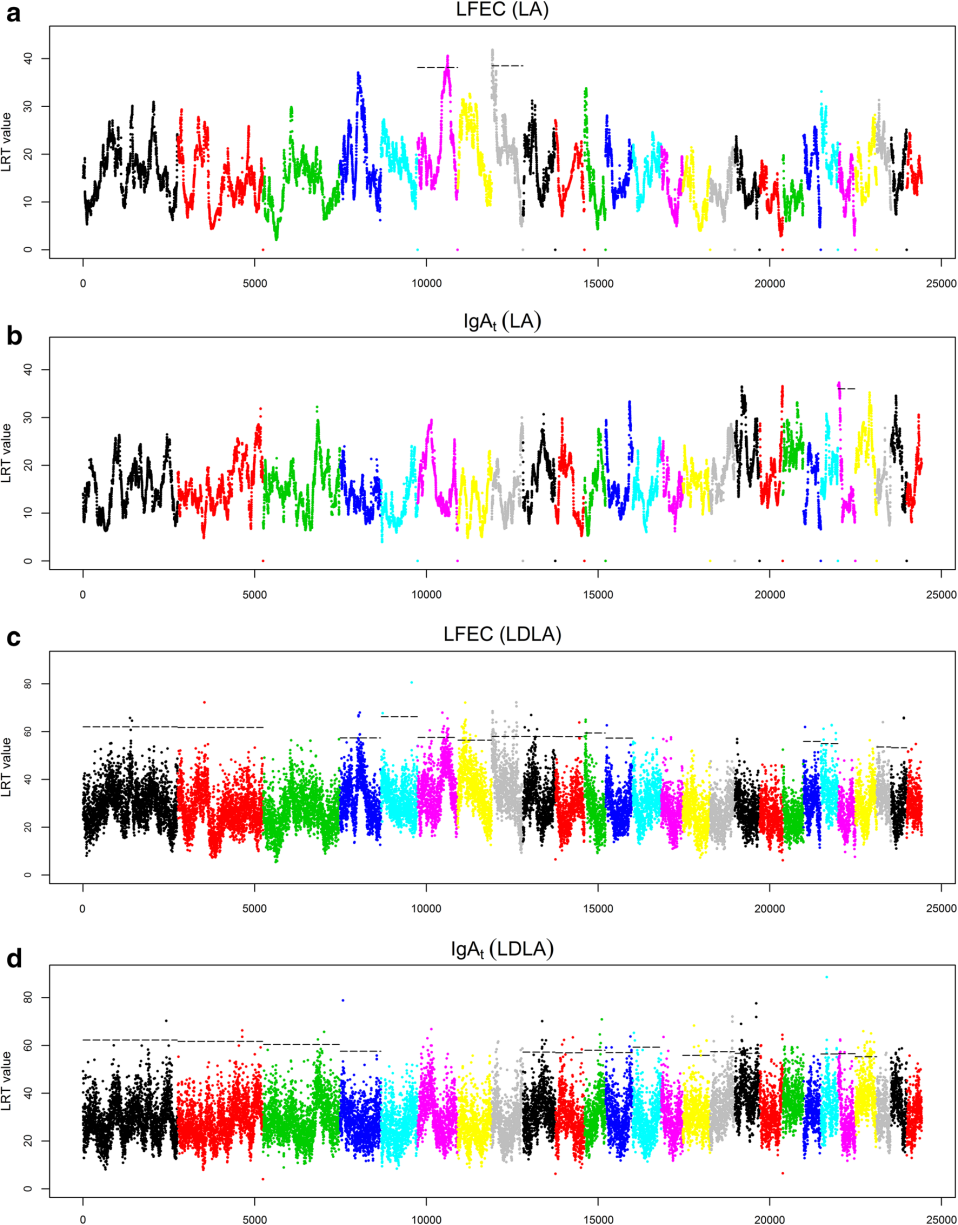
Results of linkage analysis (LA; **a**, **b**) and combined linkage disequilibrium and linkage analysis (LDLA; **c**, **d**) genome scans performed for the two indicator traits of parasite resistance analyzed. Analyzed traits: *LFEC* Log-transformed faecal egg count, *IgA*_*t*_ Box-Cox-transformed optical density ratio (ODR) values of immunoglobulin A activity. Likelihood ratio test (LRT) values obtained across the 26 ovine autosomes are represented. For those chromosomes that harbor significant QTL, the* horizontal lines* indicate the 5 % chromosome-wise significance threshold for LA (**a**, **b**) and the 5 % chromosome-wise significance threshold for LDLA (**c**, **d**)

The across-family regression analysis performed for LFEC and IgA_t_ across the ovine autosomes identified three chromosome-wide significant QTL. Two of these QTL that are located on OAR6 (OAR for *Ovis aries* chromosome) (peak at 88.1 cM) and OAR8 (peak at 2 cM) had an effect on LFEC (Fig. 1a), whereas the other QTL located on OAR22 (peak at 3.4 cM) had effects on IgA_t_ (Fig. 1b).

The significant QTL identified by the across-family LA (maximum LRT value and CI estimated by the 1-LOD drop-off method), together with the results of the within-family analyses are in Table 1. The QTL for LFEC on OAR6 and OAR8 segregated in three and two families, respectively, whereas a single family was significant for the QTL for IgA_t_ on OAR22. The CI that were estimated for the individual segregating families were located in the same region as the corresponding across-family CI, except for the peak for the QTL on OAR8 of Family 4, which was located at a more central position (31.2 cM) compared to the across-family peak at the proximal end of OAR8 (2 cM). However, the statistical profile for this family displayed a second peak reaching the 5 % chromosome-wise significance threshold (LRT = 11.76) at 12 cM, which was closer to the across-family QTL peak. The QTL effects estimated for the individual sires ranged from 0.3 (for the QTL for LFEC on OAR6) to 0.78 (for the QTL for LFEC on OAR8) standard deviations (Table 1). The estimated proportions of phenotypic variance explained by the three QTL identified by the LA were very similar and small (0.075, 0.077 and 0.069 % for the QTL on OAR6, 8 and 22, respectively).

#### LDLA results

Sixty-three significant QTL were detected at the 5 % chromosome-wise significance level by LDLA (30 for LFEC and 33 for IgA_t_). Among these 63 QTL, 13 (six for LFEC and seven for IgA_t_) reached the 5 % genome-wise significance level (Table 2; Fig. 1d). For 37 of the significant LDLA associations, nearby significant positions were grouped within a significant LDLA interval (Table 2); the remaining significant QTL identified by LDLA were defined based on isolated significant haplotypes. In addition, the three significant QTL identified by LA (on OAR6, 8 and 22) were supported by the LDLA scan (Table 2) (see Additional file 2: Figure S1). On OAR6, the LDLA results for LFEC revealed two 5 % chromosome-wise significant associations at 36 and 89.9 cM, with the latter being included within the CI of the QTL for LFEC on OAR6 detected by LA (Table 2). This analysis also identified a genome-wise significant association within the interval between 72.3 and 77.2 cM on OAR6.

On OAR8, although the LDLA scan identified a significant association at the proximal end of the chromosome (between 0.3 and 12.8 cM), which corresponded to the across-family CI for the QTL identified by LA, four other significant haplotype associations were identified across the chromosome (Table 2). Coincident with the QTL for IgA_t_ on OAR22 detected by LA (between 0.3 and 5.8 cM), the LDLA scan revealed a chromosome-wise significant haplotype association (maximum LRT at 6.7 cM) at the proximal end of this chromosome.

#### LDA results

For the three chromosomes for which the QTLMap LDA approach was implemented, several 5 % chromosome-wise significant associations were identified for the same trait for which significant results were observed in the LA and LDLA (See Additional file 1: Table S1). A correspondence was found between the significant LDA association of the 75.8–85.1 cM region on OAR6 with LFEC and the LA and LDLA results. The other significant associations identified by LDA coincided with QTL detected by LDLA.

#### GWAS results

**Fig. 2:**
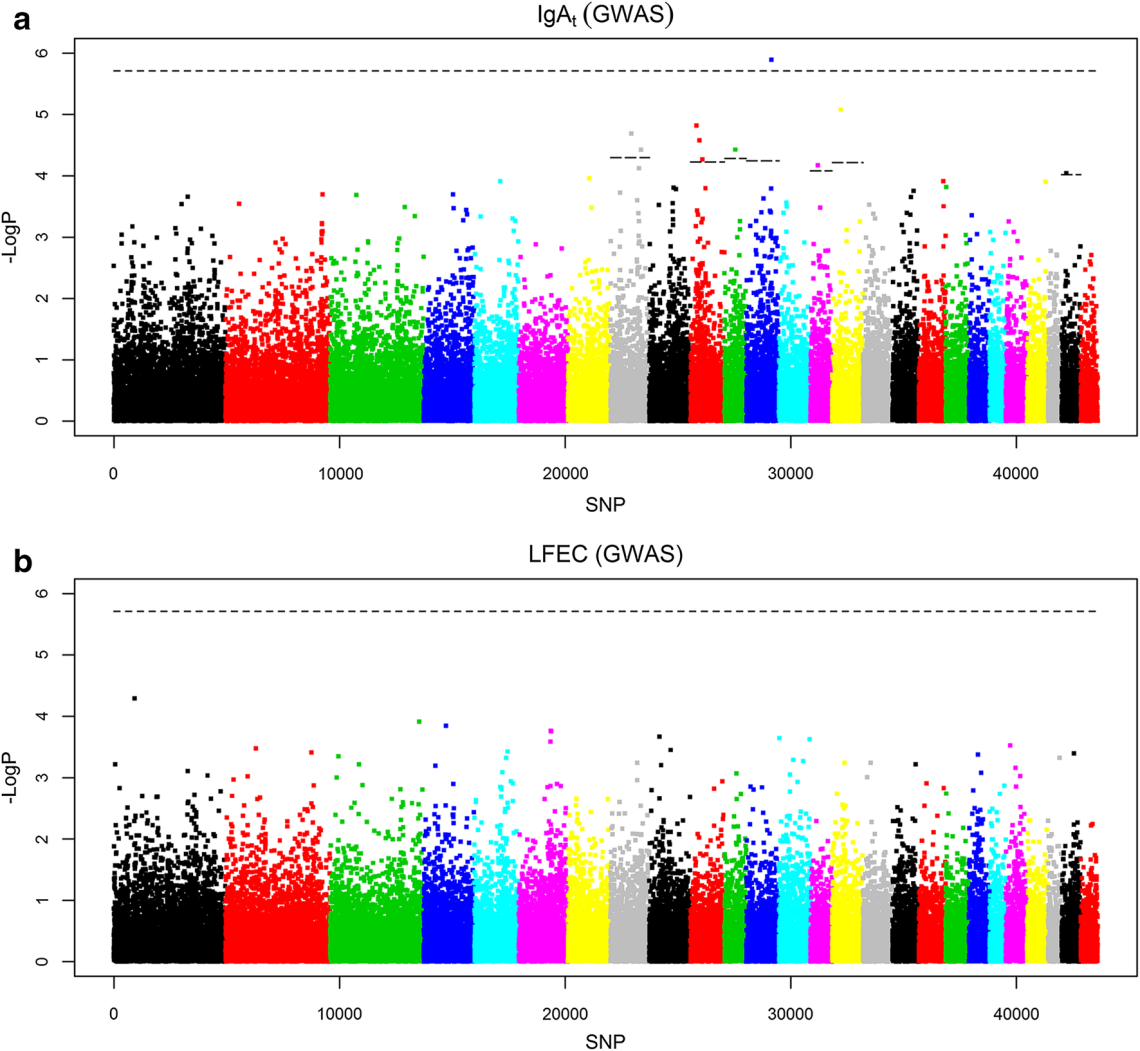
Results from the genome-wide association study (GWAS) performed for the two indicator traits of parasite resistance analyzed. Analyzed traits: *LFEC* Log-transformed faecal egg count, *IgA*_*t*_ Box-Cox-transformed optical density ratio (ODR) values of immunoglobulin A activity. The values of the log(1/P-value) are shown for all the 43,613 SNPs that passed the quality control. For the chromosomes that harbor significant SNP associations, the* horizontal lines* indicate the 5 % chromosome-wise significance threshold obtained by applying a Bonferroni correction considering the number of independent SNPs analyzed for each chromosome. The genome-wise significance threshold, considering the number of independent markers analyzed for the entire genome is also represented

None of the analyzed SNPs reached significance for LFEC (Table 3; Fig. 2a). For IgA_t_, the GWAS identified one 5 % genome-wise significant SNP on OAR12 and nine additional 5 % chromosome-wise significant associations that were distributed on six chromosomes (OAR8, 10, 11, 14, 15 and 25) (Table 3; Fig. 2b). The allelic substitution effect of the significant SNPs identified for IgA_t_ ranged from 0.243 to 0.417 phenotypic SD units. Although more than one significant SNP was identified on OAR8 and 10, these SNPs were located at relatively large distances on the chromosome (i.e., 22.8 and 13.9 Mb, respectively). Among the ten significant GWAS associations reported here for IgA_t_, one located on OAR10 was coincident with a significant QTL identified by LDLA for the same trait (between 21.5 and 27.2 cM), whereas two other associations, located on OAR8, overlapped with QTL for LFEC identified by LDLA.

### Correspondence of the detected associations with previously reported QTL for parasite resistance traits

The QTL for parasite resistance traits previously reported in sheep that coincide with the TGI reported here and are associated with the significant QTL and SNP associations identified here are summarized in Additional file 3: Table S2. Overall, we found correspondences with other studies for half of the 76 significant QTL identified by the three genome scans performed in this study.

### List of functional candidate genes

A total of 905 unique genes were extracted from the TGI that were defined for the significant QTL detected by LA, LDLA and GWAS (416 and 489 unique genes extracted from FEC- and IgA_t_-associated regions, respectively) (see Additional file 4: Table S3). From the list of 5029 known immune-related genes, we performed a survey for positional candidate genes and identified 205 functional candidate genes (indicated in blue font in Additional file 4: Table S3), which were all extracted from TGI related to significant QTL that were detected by LA or LDLA. Gene symbols of these functional candidate genes are in Tables 1 and 2 based on their genomic locations within the corresponding QTL regions.

## Discussion

The genetic architecture of resistance to internal parasites is a complex trait that is influenced by many loci with small effects [21]. Using two different approaches to correct for sampling errors associated with single-marker regression, Kemper et al. [21] estimated that the largest effects that influence fecal worm egg count for *Trichostrongylus colubriformis* explained between 0.12 and 0.48 % of the phenotypic variance. These authors suggest that such small effects are shared by many complex traits and are not specific to parasite resistance. The proportions of phenotypic variance explained by the significant LA associations reported here, which were equal to ~0.074 %, are slightly lower than the lower limit of the range reported by Kemper et al. [21], although the estimated effects are within the ranges reported in other related studies [19, 20, 22]. Considering the small size of the targeted genetic effects to be detected, the statistical power of QTL detection for indicators of parasite resistance may be limited in such experiments if the number of sampled individuals is not very large. Based on Weller et al. [47], we estimated that the statistical power of QTL detection for QTL with a substitution effect of 0.2 phenotypic SD units, two alleles with frequencies of 0.25 and 0.75, respectively, and for a trait with a heritability of 0.2 (considering the estimates of Gutiérrez-Gil et al. [48]) was approximately 11 %. This estimate is based on the following assumptions i.e. (1) a type I error rate of 0.05, (2) a 1 % recombination frequency between the QTL and SNP and (3) 37.5 % of the analyzed sires are heterozygous at the QTL.

Our study successfully identified QTL that influence the two indicator traits related to GIN resistance using LA and LDLA, whereas the GWAS analysis only detected significant SNP associations with IgA_t_. The different analyses performed in this study can detect significant associations with different features. Hence, because classical LA will only detect QTL in our design if several sires are heterozygous at the same QTL (*Qq*), many marker-trait associations that do not satisfy this assumption but have a genuine association at the population level, will not be detected by LA; however, such associations can be detected by either of the two alternative genome scan analyses performed here i.e. LDLA or GWAS. Therefore, we attempted to present a global picture of the associations that segregate in this commercial sheep population by complementing the limits of classical LA with these alternative LDLA and GWAS approaches, which exploit population information. In our case, the GWAS approach also identified a substantially lower number of associations than LDLA. This may be explained by the fact that modeling both the association (LD) and the transmission (linkage) in a single analysis, LDLA permits to map QTL more accurately than LA while retaining its robustness to spurious associations [40]. In addition, among the different advantages highlighted for the use of LDLA versus GWAS for animal populations, Meuwissen et al. [49] claimed that LDLA is expected to suffer less from multiple-testing, and therefore to have more power to detect the existing QTL.

For the chromosomes that showed coincident significant results identified by LA and LDLA, we performed an exploratory LDA analysis with the QTLMap software (see Additional file 1: Table S1, Additional file 2: Figure S1). This analysis differs from GWAS in that parental haplotypes are pooled in classes that are defined by the identity-by-state (IBS) status of the haplotypes, with each different haplotype class having a specific effect on the quantitative trait [40]. The significant LDA results obtained for OAR6, 8 and 22 supported several of the significant LDLA associations reported for these chromosomes; whereas the LDA result obtained for OAR6 at 85.1 Mb supported the significant QTL that was detected by both LA and LDLA. This observation strengthens the support for the QTL for LFEC identified by LA on OAR6, which suggests that in addition to a family-based linkage information signal, the effect is also due to a genuine association with the trait, although it was not identified in our GWAS (most likely as a consequence of the limited power of the experimental design).

Regarding the LFEC-related results for OAR6 that were obtained by LA, LDLA and LDA, in the current study, we replicated the most significant QTL that was previously identified through a microsatellite-based genome scan using a different set of Churra sheep half-sib families [20]. In the latter study, the peak of the genome-wise significant QTL for LFEC was located in the marker interval BM4621-CSN3 on OAR6, which corresponds to a region between 68 and 85.1 Mb in the current sheep genome assembly (Oar_v3.1). The mentioned flanking interval overlaps with the TGI defined here for LFEC on OAR6 by LA (between 80.8 and 91.4 Mb) (Table 1), LDLA (between 72.3 and 77.2 and between 85 and 90.2 Mb) (Table 2) and LDA (between 75.8 and 77.7 and between 85 and 85.1 Mb) (see Additional file 1: Table S1, Additional file 2: Figure S1). This finding provides support for the design and planning of future fine-mapping studies for this chromosomal region. The higher marker density and information provided by the complementary analyses reported here for this region suggest that the OAR6 region ranging from 68 to 91.4 Mb includes several different QTL that directly influence GIN resistance in Churra sheep. Interestingly, a GWAS on a Red Maasai x Dorper backcross sheep population [26] also suggested the presence of several QTL for FEC in lambs within a region between 55.9 and 78.19 Mb on OAR6. This finding was based on the fact that the most significant SNP association with FEC identified on OAR6 at 74.86 Mb, was proven not to be in LD with nearby clusters of significant markers for the same trait (in intervals between 55.9 and 62.6 Mb, 74.1 and 75.00 Mb, and 78.1 and 78.2 Mb) (see Additional file 3: Table S2). In spite of the remarkable correspondence between these results and our results, the most distal signals that were detected on OAR6 in our study (TGI defined by LA: 80.8 to 91.4 Mb; LDLA: 85 to 90.2 Mb; and LDA: 85 to 85.1 Mb) do not overlap with any previously reported QTL in other populations, but only with those previously reported by Gutiérrez-Gil et al. [20] (see Additional file 3: Table S2) . With the exception of Gutiérrez-Gil et al. [20] work, most studies refer to QTL that are detected for young animals (lambs); thus, the most distal QTL that we identified on OAR6 could be related to specific mechanisms of the immune response that is activated in adult animals. As suggested by Stear et al. [50], the genetic variation in fecal egg counts in lambs is a consequence of genetic variation in worm length and hence worm fecundity; in contrast, mature sheep may be able to regulate both fecundity and worm number. These authors suggested that the lower fecal egg counts observed in adult animals compared to lambs are due to the acquisition of effective immune responses that reduce worm numbers, possibly via immediate hypersensitivity reactions against incoming third-stage larvae [51]. Recent studies have highlighted differences in the pathways involved in innate and acquired resistance [52]. Another correspondence that was observed with the results reported by Gutiérrez-Gil et al. [20] concerned the QTL for LFEC detected by LDLA on OAR10 (TGI: 70.01–71.55 Mb) (see Additional file 3: Table S2). Due to the lack of evidence from the other analyses reported here, this region was not further investigated.

An intriguing finding is that the other two QTL detected by LA in this work did not coincide with QTL that were reported for other sheep populations, whereas three of the ten significant SNP associations identified by GWAS, and 35 of the 63 significant QTL identified by LDLA, overlapped with QTL effects described in other studies (see Additional file 3: Table S2). Indeed, the significant GWAS results coincided with QTL on OAR8 reported by Crawford et al. [13] and Silva et al. [19], on OAR12 by Riggio et al. [24], and on OAR15 by Silva et al. [19] (see Additional file 3: Table S2). In our study, the SNP association on OAR12 at 61.9 Mb was the only one that reached the 5 % genome-wide significance level. Although not mentioned in Additional file 3: Table S2 because there was no complete overlap, Beh et al. [12] used microsatellite markers to identify a QTL in this genomic region (between 63.5 and 71.5 Mb) for FEC-related traits in *T. colubriformis* infection. It should be noted that we did not find a clear correspondence with the classical regions reported to influence parasite resistance traits, such as those that harbor the ovine *IFN*-*γ* gene (OAR3: 151.53 Mb) [11, 14, 17] or the major histocompatibility complex-related genes (OAR20: 7 Mb; 24–26 Mb; 58–60 Mb) [14].

Among the large number of correspondences between our LDLA results and previously reported studies (see Additional file 3: Table S2), those that are based on data from the 50 K chip are of special relevance because of the proximity between the QTL peaks reported here and in other studies. Apart from the correspondences with the findings of Benavides et al. [26] mentioned above for OAR6, those found for the QTL on OAR5 (TGI: 89.68–90.14 Mb) are particularly relevant. This QTL identified by LDLA is located in a region where several significant effects for a wide range of parasite indicator traits were reported by Sallé et al. [22], which suggests the presence of a QTL with pleiotropic effects.

We identified 205 immune-related genes within the TGI defined by the LA and LDLA (Tables 1, 2) but none of these functional candidate genes were found in the significant GWAS-defined TGI. Some of these immune-related genes are involved in the T helper (Th) 2 cell response, which orchestrates the mechanisms of tissue repair as a primary host defense against helminthes [53], whereas others are linked to the Th1 cell response, which is associated with progression to chronic infection [54].

Due to the large number of significant regions identified and the need for additional fine-mapping results to propose reliable promising causal candidate genes, in the following part, we only discuss below the genes that were identified in relation to the QTL for LFEC identified by LA on OAR6 (TGI: 80.9–91.4 Mb), which include the genes extracted for the LDLA-defined TGI between 85 and 90.2 Mb. The fact that this QTL, previously reported by Gutiérrez-Gil et al. [20], was also identified for the population analyzed here and the support provided by the related signals identified by LDLA/LDA, led us to carry out a preliminary assessment of the 20 positional candidate immune-related genes that map to this region (Table 1). Among these genes, several encode chemokines (IL8, CXCL1, CXCL10, CXCL11, CXCL9, PF4, PPBP), a family of small proteins that play important roles in the immune system through leukocyte recruitment, cell communication and cell activation during infection [55, 56]. In particular, IL8 (or CXCL8) and CXCL1 are involved in the recruitment and activation of neutrophils [55]. IL8 also participates in the recruitment of mast cells, which are frequently associated with the Th2 cell response [57]. CXCL9, CXCL10 and CXCL11, which are induced by IFN-γ, are involved in promoting the Th1 immune response. In nematode-infected mice, CXCL10 slows down the intestinal epithelial cell turnover rate and thus, increases worm survival [58]. In addition, both PF4 and PPBP have been suggested to play roles in wound healing [59, 60]. Three genes coding for members of the epidermal growth factor family also map to the considered region on OAR6: *AREG* (*amphiregulin*), *BTC* (*betacellulin*) and *EREG* (*epiregulin*). *AREG* is expressed by diverse cell types involved in the immune response, such as activated Th2 cells [61], and is a central mediator of epithelial repair [62]. In mice, lack of *AREG* expression appears to have an effect on the delayed expulsion of GIN [63]. Because wound repair and GIN expulsion are related to the acquired Th2 response [53, 64], genes associated with these mechanisms (e.g., *IL8*, *PF4*, *PPBP* and *AREG*) could be of interest when searching for candidates to explain an adult-specific QTL, such as the QTL detected on OAR6 between 80.8 and 91.4 Mb.

The large number of QTL identified in this study supports the idea that disease susceptibility is not determined by individual genes acting alone but rather by complex multi-gene interactions [65, 66]. Our results are the first steps towards the identification of allelic variants that directly control the phenotypic variation observed for parasite resistance in adult Churra sheep. The identification of causal variants, or SNPs in strong LD with the casual variants, could contribute to the implementation of these results in breeding schemes for the Churra breed population. Future studies that combine genomic variation analysis and functional genomic information may help to elucidate the biology of resistance to GIN diseases in sheep.

## Conclusions

In summary, the 50 K chip was used for a medium marker density scan of the sheep genome to identify regions that influence traits related to resistance to GIN infections in adult animals. By exploiting the information obtained at the within-family level and at the population level, three methods of analysis were used (LA, LDLA and GWAS) to provide a global picture of the QTL that segregate in the commercial population of Churra sheep analyzed. Many of the significant associations reported here overlap with previously reported QTL for different populations of young sheep. These results will contribute to identify target regions that control variation of the complex parasite resistance trait in sheep, independently of the age of the animals. Other significant associations that did not coincide with previously reported QTL could be related to the specific immune response of adult animals. This study also replicated a QTL for FEC on OAR6 that was previously reported in a different subset of animals from the commercial population of Churra sheep. Together, the enhanced marker density provided by the 50 K chip and the complementary analyses reported here suggest that several QTL are present in this genomic region. This replication and the re-definition of these genetic effects in the independent population analyzed here provide support for investing future research efforts aimed at identifying the corresponding causal allelic variants. The combination of high-density SNP genotyping (700 K SNP array) and whole-genome sequencing of segregating trios (composed by a segregating sire carrying the *Qq* genotype, and two homozygous daughters for alternative haplotype alleles, *QQ* and *qq*, and showing extreme divergence for the resistance phenotype) could be a powerful strategy to reach this objective.

## Additional files

10.1186/s12711-016-0182-4 Chromosome-wise significant results (Pc-value < 0.05) identified by the linkage disequilibrium analysis (LDA) performed in the present study for chromosomes (OAR) 6, 8 and 22. Characterization of the chromosome-wise significant results (Pc-value < 0.05) identified by the QTLMap linkage disequilibrium analysis (LDA) that was performed for the three chromosomes showing coincident results in the LA and LDLA genome scans presented here for parasite resistance traits.
10.1186/s12711-016-0182-4 Profiles of the Likelihood Ratio Test (LRT) obtained from the linkage analysis (LA), linkage disequilibrium analysis (LDA) and the combined LDLA performed for chromosomes (OAR) 6 (a; LFEC), 8 (b; LFEC), and 22 (c; IgA_t_). For the indicated trait, the LRT results of LA (solid line), LDA (dark gray circle), and LDLA (light gray circle) (y-axis) are plotted against the SNP positions analyzed along chromosomes (OAR) 6, 8 and 22 (x-axis). The 5 % chromosome-wise significance thresholds considered for each of three analyses are represented as horizontal lines.
10.1186/s12711-016-0182-4 Summary table of the correspondence between the QTL and SNP associations identified in the present study and other studies previously reported for parasite resistance traits in sheep. This table shows the correspondences found for all the QTL identified in this study (by LA, LDLA and GWAS) (indicated in green cells) with QTL previously reported based on microsatellite-based studies (compiled in the SheepQTLdb; indicated in light orange cells) and SNP chip-based studies (indicated in orange cells).
10.1186/s12711-016-0182-4 Total list of annotated genes extracted from the Sheep Genome Assembly v3.1 using the BioMart web-tool for the significant QTL regions and SNP associations identified for the two parasite resistance traits analyzed in the present study. Among the total list of genes extracted, we identified 205 functional candidate genes involved in the immune response, based on our candidate gene survey, which are indicated in blue font colour. The colour of the rows refer to genes extracted based on the results of the Linkage Analysis (LA; green), Combined Linkage Disequilibrium and Linkage Analysis (LDLA; yellow) and Genome-wise Association Study (GWAS; blue).

## References

[1] Kaplan RM, Vidyashankar AN (2012). An inconvenient truth: global worming and anthelmintic resistance. Vet Parasitol.

[2] Raadsma HW, Gray GD, Woolaston RR. Genetics of disease resistance and vaccine response. In: Piper L, Ruvinsky A, editors. The Genetics of Sheep. University Press: Cambridge; 1997. p. 199–224.

[3] Stear MJ, Doligalska M, Donskow-Schmelter K (2007). Alternatives to anthelmintics for the control of nematodes in livestock. Parasitology.

[4] Morris CA, Wheeler M, Watson TG, Hosking BC, Leathwick DM (2005). Direct and correlated responses to selection for high or low faecal nematode egg count in Perendale sheep. NZJ Agric Res.

[5] Karlsson LJE, Greeff JC (2006). Selection response in fecal worm egg counts in the Rylington Merino parasite resistant flock. Aust J Exp Agric.

[6] Kemper KE, Elwin RL, Bishop SC, Goddard ME, Woolaston RR (2009). *Haemonchus contortus* and *Trichostrongylus colubriformis* did not adapt to long-term exposure to sheep that were genetically resistant or susceptible to nematode infections. Int J Parasitol.

[7] Grencis RK (2015). Immunity to helminths: resistance, regulation, and susceptibility to gastrointestinal nematodes. Annu Rev Immunol.

[8] Jiang Y, Xie M, Chen W, Talbot R, Maddox JF, Faraut T (2014). The sheep genome illuminates biology of the rumen and lipid metabolism. Science.

[9] Hein WR, Pernthaner A, Piedrafita D, Meeusen EN (2010). Immune mechanisms of resistance to gastrointestinal nematode infections in sheep. Parasite Immunol.

[10] Hu ZL, Park CA, Wu XL, Reecy JM (2013). Animal QTLdb: an improved database tool for livestock animal QTL/association data dissemination in the post-genome era. Nucleic Acids Res.

[11] Coltman DW, Wilson K, Pilkington JG, Stear MJ, Pemberton JM (2001). A microsatellite polymorphism in the gamma interferon gene is associated with resistance to gastrointestinal nematodes in a naturally-parasitized population of Soay sheep. Parasitology.

[12] Beh KJ, Hulme DJ, Callaghan MJ, Leish Z, Lenane I, Windon RG (2002). A genome scan for quantitative trait loci affecting resistance to *Trichostrongylus colubriformis* in sheep. Anim Genet.

[13] Crawford AM, Paterson KA, Dodds KG, Diez Tascon C, Williamson PA, Roberts Thomson M (2006). Discovery of quantitative trait loci for resistance to parasitic nematode infection in sheep: I. Analysis of outcross pedigrees. BMC Genomics.

[14] Davies G, Stear MJ, Benothman M, Abuagob O, Kerr A, Mitchell S (2006). Quantitative trait loci associated with parasitic infection in Scottish blackface sheep. Heredity (Edinb).

[15] Beraldi D, McRae AF, Gratten J, Slate J, Visscher PM, Pemberton JM (2007). Mapping quantitative trait loci underlying fitness-related traits in a free-living sheep population. Evolution.

[16] Marshall K, Maddox JF, Lee SH, Zhang Y, Kahn L, Graser HU (2009). Genetic mapping of quantitative trait loci for resistance to *Haemonchus contortus* in sheep. Anim Genet.

[17] Dominik S, Hunt PW, McNally J, Murrell A, Hall A, Purvis IW (2010). Detection of quantitative trait loci for internal parasite resistance in sheep. I. Linkage analysis in a Romney × Merino sheep backcross population. Parasitology.

[18] Matika O, Pong-Wong R, Woolliams JA, Bishop SC (2011). Confirmation of two quantitative trait loci regions for nematode resistance in commercial British terminal sire breeds. Animal.

[19] Silva MV, Sonstegard TS, Hanotte O, Mugambi JM, Garcia JF, Nagda S (2012). Identification of quantitative trait loci affecting resistance to gastrointestinal parasites in a double backcross population of Red Maasai and Dorper sheep. Anim Genet.

[20] Gutierrez-Gil B, Perez J, Alvarez L, Martinez-Valladares M, de la Fuente LF, Bayon Y (2009). Quantitative trait loci for resistance to trichostrongylid infection in Spanish Churra sheep. Genet Sel Evol..

[21] Kemper KE, Emery DL, Bishop SC, Oddy H, Hayes BJ, Dominik S (2011). The distribution of SNP marker effects for faecal worm egg count in sheep, and the feasibility of using these markers to predict genetic merit for resistance to worm infections. Genet Res (Camb)..

[22] Sallé G, Jacquiet P, Gruner L, Cortet J, Sauvé C, Prévot F (2012). A genome scan for QTL affecting resistance to *Haemonchus contortus* in sheep. J Anim Sci.

[23] Riggio V, Matika O, Pong-Wong R, Stear MJ, Bishop SC (2013). Genome-wide association and regional heritability mapping to identify loci underlying variation in nematode resistance and body weight in Scottish Blackface lambs. Heredity (Edinb)..

[24] Riggio V, Pong-Wong R, Sallé G, Usai MG, Casu S, Moreno CR (2014). A joint analysis to identify loci underlying variation in nematode resistance in three European sheep populations. J Anim Breed Genet.

[25] McRae KM, McEwan JC, Dodds KG, Gemmell NJ (2014). Signatures of selection in sheep bred for resistance or susceptibility to gastrointestinal nematodes. BMC Genomics.

[26] Benavides MV, Sonstegard TS, Kemp S, Mugambi JM, Gibson JP, Baker RL (2015). Identification of novel loci associated with gastrointestinal parasite resistance in a Red Maasai × Dorper backcross population. PLoS One.

[27] García-Pérez AL, Hurtado A, Oregui LM, Juste RA. Effects of a second annual strategic anthelmintic treatment in dairy sheep in Northern Spain. Small Rumin Res. 2002;43:121–6.

[28] Houdijk JGM, Kyriazakis I, Coop RL, Jackson F (2001). The expression of immunity to *Teladorsagia circumcincta* in ewes and its relationship to protein nutrition depend on body protein reserves. Parasitology.

[29] Wolstenholme AJ, Fairweather I, Prichard R, von Samson-Himmelstjerna G, Sangster NC (2004). Drug resistance in veterinary helminths. Trends Parasitol.

[30] Fish and Food Ministry of Agriculture. Manual of veterinary parasitological laboratory techniques. 1986.

[31] Martinez-Valladares M (2005). Vara-Del Rio MP, Cruz-Rojo MA, Rojo-Vazquez FA. Effect of a low protein diet on the resistance of Churra sheep to *Teladorsagia circumcincta*. Parasite Immunol.

[32] Fox J, Weisberg S, Bates D, Fox MJ (2012). Package ‘car’.

[33] R Core Team. R: A language and environment for statistical computing. R Foundation for Statistical Computing, Vienna. 2014. http://www.R-project.org/.

[34] García-Gámez E, Gutiérrez-Gil B, Sahana G, Sánchez JP, Bayón Y, Arranz JJ (2012). GWA analysis for milk production traits in dairy sheep and genetic support for a QTN influencing milk protein percentage in the *LALBA* gene. PLoS One.

[35] The CSIRO Animal, Food and Health Sciences livestock genomics web site. CSIRO Australia. The *Ovis aries* reference genome assembly (http://www.livestockgenomics.csiro.au/sheep/oar3.1.php). Accessed 21 Jan 2013.

[36] Lenth Russell V. lsmeans: least-squares means. R package version 1.06–05. 2013.

[37] Filangi O, Moreno C, Gilbert H, Legarra A, Le Roy P, Elsen J. QTLMap, a software for QTL detection in outbred populations. In Proceedings of the 9th World Congress on Genetics Applied to Livestock Production: 1–6 August 2010; Leipzig; 2010.

[38] Lander ES, Botstein D (1989). Mapping mendelian factors underlying quantitative traits using RFLP linkage maps. Genetics.

[39] Broman KW, Sen S. A Guide to QTL Mapping with R/qtl (Vol. 46). New York: Springer; 2009.

[40] Legarra A, Fernando RL (2009). Linear models for joint association and linkage QTL mapping. Genet Sel Evol..

[41] Madsen P, Sørensen P, Su G, Damgaard LH, Thomsen H, Labouriau R. DMU-a package for analyzing multivariate mixed models. In Proceedings of the 8th World Congress on Genetics Applied to Livestock Production: 13–18 August 2006; Belo Horizonte. 2006.

[42] Gao X, Becker LC, Becker DM, Starmer JD, Province MA (2010). Avoiding the high Bonferroni penalty in genome-wide association studies. Genet Epidemiol.

[43] simpleM. A multiple testing correction program for correlated SNPs. (https://dsgweb.wustl.edu/rgao/). Accessed 15 Nov 2014.

[44] Ensembl release 81—August 2015 WTSI/EMBL-EBI (http://www.ensembl.org/biomart/martview/). Accessed 15 Aug 2015.

[45] Kelley J, de Bono B, Trowsdale J (2005). IRIS: a database surveying known human immune system genes. Genomics.

[46] Innate Database Gene Lists: (http://www.innatedb.com/redirect.do?go=resourcesGeneLists). Accessed 15 Feb 2015.

[47] Weller JI, Kashi Y, Soller M (1990). Power of daughter and granddaughter designs for determining linkage between marker loci and quantitative trait loci in dairy cattle. J Dairy Sci.

[48] Gutiérrez-Gil B, Pérez J, de la Fuente L, Meana A, Martínez-Valladares M, San Primitivo F (2010). Genetic parameters for resistance to trichostrongylid infection in dairy sheep. Animal.

[49] Meuwissen, T. Use of whole genome sequence data for QTL mapping and genomic selection. In Proceedings of the 9th World Congress on Genetics Applied to Livestock Production: 1–6 August 2010; Leipzig; 2010.

[50] Stear MJ, Strain S, Bishop SC (1999). Mechanisms underlying resistance to nematode infection. Int J Parasitol.

[51] Stear MJ, Bishop SC, Doligalska M, Duncan JL, Holmes PH, Irvine J (1995). Regulation of egg production, worm burden, worm length and worm fecundity by host responses in sheep infected with *Ostertagia circumcincta*. Parasite Immunol.

[52] Grencis RK, Humphreys NE, Bancroft AJ (2014). Immunity to gastrointestinal nematodes: mechanisms and myths. Immunol Rev.

[53] Allen JE, Wynn TA (2011). Evolution of Th2 immunity: a rapid repair response to tissue destructive pathogens. PLoS Pathog.

[54] Else KJ, Finkelman FD, Maliszewski CR, Grencis RK (1994). Cytokine-mediated regulation of chronic intestinal helminth infection. J Exp Med.

[55] Schumacher C, Clark-Lewis I, Baggiolini M, Moser B (1992). High- and low-affinity binding of GRO alpha and neutrophil-activating peptide 2 to interleukin 8 receptors on human neutrophils. Proc Natl Acad Sci USA.

[56] Trotta T, Costantini S, Colonna G (2009). Modelling of the membrane receptor CXCR3 and its complexes with CXCL9, CXCL10 and CXCL11 chemokines: putative target for new drug design. Mol Immunol.

[57] da Silva EZ, Jamur MC, Oliver C (2014). Mast cell function: a new vision of an old cell. J Histochem Cytochem.

[58] Cliffe LJ, Humphreys NE, Lane TE, Potten CS, Booth C, Grencis RK (2005). Accelerated intestinal epithelial cell turnover: a new mechanism of parasite expulsion. Science.

[59] Senior RM, Griffin GL, Huang JS, Walz DA, Deuel TF (1983). Chemotactic activity of platelet alpha granule proteins for fibroblasts. J Cell Biol.

[60] Dvonch VM, Murphey RJ, Matsuoka J, Grotendorst GR (1992). Changes in growth factor levels in human wound fluid. Surgery.

[61] Zaiss DM, van Loosdregt J, Gorlani A, Bekker CP, Gröne A, Sibilia M (2013). Amphiregulin enhances regulatory T cell-suppressive function via the epidermal growth factor receptor. Immunity.

[62] Monticelli L, Sonnenberg G, Abt M, Osborne L, Wojno ET, Alenghat T (2013). Innate lymphoid cells promote airway epithelial repair through the amphiregulin-EGFR pathway (P3250). J Immunol.

[63] Zaiss DM, Yang L, Shah PR, Kobie JJ, Urban JF, Mosmann TR (2006). Amphiregulin, a TH2 cytokine enhancing resistance to nematodes. Science.

[64] Allen JE, Sutherland TE (2014). Host protective roles of type 2 immunity: parasite killing and tissue repair, flip sides of the same coin. Semin Immunol.

[65] Kadarmideen HN, Watson-Haigh NS, Andronicos NM (2011). Systems biology of ovine intestinal parasite resistance: disease gene modules and biomarkers. Mol BioSyst.

[66] Moore JH (2003). The ubiquitous nature of epistasis in determining susceptibility to common human diseases. Hum Hered.

