# Supplementary material for: Detection and replication of QTL underlying resistance to gastrointestinal nematodes in adult sheep using the ovine 50K SNP array
Source: Genet Sel Evol. 2016 Jan 20;48:4. doi: 10.1186/s12711-016-0182-4 (PMC4719203; doi:10.1186/s12711-016-0182-4)
Supplement: Supplementary file 1 — 10.1186/s12711-016-0182-4 Chromosome-wise significant results (Pc-value < 0.05) identified by the linkage disequilibrium analysis (LDA) performed in the present study for chromosomes (OAR) 6, 8 and 22. Characterization of the chromosome-wise significant results (Pc-value < 0.05) identified by the QTLMap linkage disequilibrium analysis (LDA) that was performed for the three chromosomes showing coincident results in the LA and LDLA genome scans presented here for parasite resistance traits. [file 12711_2016_182_MOESM1_ESM.doc]

**Additional file 1 Table S1: Atlija et al. Detection and replication of QTL underlying resistance to gastrointestinal nematodes in adult sheep using an ovine 50K SNP array.**

**Additional file 1 Table S1. Chromosome-wise significant results (Pc-value < 0.05) identified by the linkage disequilibrium analysis (LDA) performed in the present study for chromosomes (OAR) 6, 8 and 22.**

| OAR^1^ | Trait^2^ | Position of maximum LRT^3^ (cM) | Significant LDA interval (cM)^4^ | P_c_-value^5^ |
| --- | --- | --- | --- | --- |
| 6 | LFEC | 36.0 | 36-41.8 | <0.05 |
|  |  | 61.1 | - | <0.05 |
|  |  | 77.7 | 75.8-77.7 | <0.05 |
|  |  | 85.1 | 85-85.1 | <0.05 |
| 8 | LFEC | 37.7 | - | <0.0019 |
|  |  | 49.8 | - | <0.0019 |
|  |  | 72.1 | 64.1-72.1 | <0.0019 |
| 22 | IgA_t_ | 19.5 | - | <0.05 |
|  |  | 40.5 | 36-40.6 | <0.05 |

^1^OAR = ovine chromosome

^2^Analyzed traits: *LFEC* Log-transformed faecal egg count; *IgA_t_* Box-Cox-transformed optical density values of immunoglobulin A activity.

^3^Position of the chromosome (in centiMorgans) at which the maximum Likelihood Ration Test (LRT) is reached in the LDA performed in this work.

^4^A significant LDA interval (in centiMorgans) was defined by grouping consecutive significant 5% chromosome-wise LDA associations in a chromosome (allowing gaps no greater than 5 Mb).

^5^ P_c_-value: Chromosome-wise P-value established through 1,000 simulations.
